# Supplementary material for: Diagnostic Testing Accuracy for Helicobacter pylori Infection among Adult Patients with Dyspepsia in Cuba’s Primary Care Setting
Source: Microorganisms. 2023 Apr 11;11(4):997. doi: 10.3390/microorganisms11040997 (PMC10146794; doi:10.3390/microorganisms11040997)
Supplement: Supplementary file 1 [file microorganisms-11-00997-s001.zip › microorganisms-2252284-supplementary.pdf]

microorganisms

Supplementary Material

MDPI

Communication

Supplementary Material

Diagnostic Testing Accuracy for *Helicobacter pylori* Infection among Adult Patients with Dyspepsia in Cuba's Primary Care Setting

Amílcar Duquesne [1], Rosabel Falcón [2,*], Belkys Galindo [3], Onelkis Feliciano [2], Oderay Gutiérrez [2], Waldemar Baldoquín [3], Magile C. Fonseca [4], Rafael Llanes [2] and Luis Sarmiento [5,*]

1 Teaching Orthopedical Hospital Fructuoso Rodríguez, Havana City 10400, Cuba
2 Department of Microbiology, Center for Research, Diagnosis and Reference, "Pedro Kourí" Institute of Tropical Medicine, Havana City 11400, Cuba
3 Department of Epidemiology, Center for Research, Diagnosis and Reference, "Pedro Kourí" Institute of Tropical Medicine, Havana City 11400, Cuba
4 Department of Virology, Center for Research, Diagnosis and Reference, "Pedro Kourí" Institute of Tropical Medicine, Havana City 11400, Cuba
5 Department of Clinical Sciences, Immunovirology Unit, Skåne University Hospital, Lund University, 21428 Malmö, Sweden
* Correspondence: rosabel@ipk.sld.cu (R.F.); luis.sarmiento-perez@med.lu.se (L.S.); Tel.: +53-72553555 (R.F.); +46-703067626 (L.S.)

Keywords: *Helicobacter pylori*; diagnostic accuracy; primary care

Disclaimer/Publisher's Note: The statements, opinions and data contained in all publications are solely those of the individual author(s) and contributor(s) and not of MDPI and/or the editor(s). MDPI and/or the editor(s) disclaim responsibility for any injury to people or property resulting from any ideas, methods, instructions or products referred to in the content.

Table S1. STROBE Statement—Checklist of items that should be included in reports of cross-sectional studies

	Item No.	Recommendation	Page No.
**Title and abstract**	1	(a) Indicate the study's design with a commonly used term in the title or the abstract	1
		(b) Provide in the abstract an informative and balanced summary of what was done and what was found	1
**Introduction**			
Background/ rationale	2	Explain the scientific background and rationale for the investigation being reported	1-2
Objectives	3	State specific objectives, including any prespecified hypotheses	2
**Methods**			
Study design	4	Present key elements of study design early in the paper	2
Setting	5	Describe the setting, locations, and relevant dates, including periods of recruitment, exposure, follow-up, and data collection	2
Participants	6	(a) Give the eligibility criteria, and the sources and methods of selection of participants	2
Variables	7	Clearly define all outcomes, exposures, predictors, potential confounders, and effect modifiers. Give diagnostic criteria, if applicable	2-4
Data sources/ measurement	8*	For each variable of interest, give sources of data and details of methods of assessment (measurement). Describe comparability of assessment methods if there is more than one group	3-4
Bias	9	Describe any efforts to address potential sources of bias	3-4
Study size	10	Explain how the study size was arrived at	4
Quantitative variables	11	Explain how quantitative variables were handled in the analyses. If applicable, describe which groupings were chosen and why	-
Statistical methods	12	(a) Describe all statistical methods, including those used to control for confounding	4
		(b) Describe any methods used to examine subgroups and interactions	-
		(c) Explain how missing data were addressed	-
		(d) If applicable, describe analytical methods taking account of the sampling strategy	-
		(e) Describe any sensitivity analyses	-
**Results**			
Participants	13*	(a) Report numbers of individuals at each stage of study—e.g. numbers potentially eligible, examined for eligibility, confirmed eligible, included in the study, completing follow-up, and analysed	4
		(b) Give reasons for non-participation at each stage	4
		(c) Consider use of a flow diagram	4

Descriptive data	14*	(a) Give characteristics of study participants (e.g. demographic, clinical, social) and information on exposures and potential confounders	5
		(b) Indicate the number of participants with missing data for each variable of interest	5
Outcome data	15*	Report numbers of outcome events or summary measures	4-6
Main results	16	(a) Give unadjusted estimates and, if applicable, confounder-adjusted estimates and their precision (eg, 95% confidence interval). Make clear which confounders were adjusted for and why they were included	4-6
Other analyses	17	Report other analyses done—e.g. analyses of subgroups and interactions, and sensitivity analyses	-
**Discussion**			
Key results	18	Summarise key results with reference to study objectives	6-8
Limitations	19	Discuss the limitations of the study, taking into account sources of potential bias or imprecision. Discuss both the direction and magnitude of any potential bias	7
Interpretation	20	Give a cautious overall interpretation of results considering objectives, limitations, multiplicity of analyses, results from similar studies, and other relevant evidence	8
Generalisability	21	Discuss the generalisability (external validity) of the study results	-
**Other information**			
Funding	22	Give the source of funding and the role of the funders for the present study and, if applicable, for the original study on which the present article is based	8

Table S2. Report of Diagnostic Tests versus *H. pylori* status for each patient enrolled in the study.

Patient'data			Index Diagnostic Test				Gold standard test
ID	Age	Sex	RUT[a]	RT-PCR[b]	*H. pylori* - IgG ELISA[c]	WB[d]	*H. pylori* status[e]
1	58	M	Positive	Positive	Positive	Positive	Positive
2	54	F	Negative	Negative	Negative	Negative	Negative
3	73	F	Positive	Positive	Positive	Positive	Positive
4	39	M	Positive	Positive	Positive	Positive	Positive
5	36	M	Negative	Positive	Negative	Negative	Negative
6	50	M	Positive	Positive	Positive	Positive	Positive
7	61	F	Positive	Positive	Positive	Positive	Positive
8	57	F	Positive	Positive	Positive	Positive	Positive
9	50	F	Negative	Positive	Positive	Positive	Negative
10	66	M	Negative	Positive	Positive	Positive	Negative
11	28	F	Negative	Positive	Negative	Positive	Negative
12	42	M	Negative	Negative	Negative	Negative	Negative
13	45	F	Positive	Positive	Positive	Positive	Positive
14	50	F	Positive	Positive	Positive	Positive	Positive
15	61	F	Negative	Negative	Negative	Negative	Negative
16	55	F	Negative	Negative	Positive	Negative	Negative
17	47	M	Negative	Positive	Positive	Positive	Negative
18	71	F	Positive	Positive	Positive	Positive	Positive
19	23	F	Negative	Negative	Negative	Negative	Negative
20	52	F	Positive	Positive	Positive	Positive	Positive
21	68	F	Positive	Positive	Positive	Positive	Positive
22	67	F	Negative	Negative	Positive	Negative	Negative
23	24	M	Negative	Negative	Negative	Negative	Negative
24	49	M	Positive	Positive	Positive	Positive	Positive
25	43	M	Negative	Negative	Negative	Negative	Negative
26	40	F	Positive	Positive	Positive	Positive	Positive
27	57	F	Negative	Negative	Negative	Negative	Negative
28	33	F	Positive	Positive	Positive	Positive	Positive
29	53	F	Positive	Positive	Positive	Positive	Positive
30	25	M	Negative	Positive	Negative	Positive	Negative
31	46	F	Negative	Positive	Negative	Positive	Negative
32	69	M	Positive	Negative	Positive	Negative	Positive
33	18	F	Negative	Negative	Negative	Negative	Negative
34	52	M	Negative	Negative	Negative	Negative	Negative
35	49	M	Positive	Positive	Positive	Positive	Positive
36	24	F	Negative	Negative	Positive	Negative	Negative
37	59	F	Positive	Negative	Positive	Positive	Positive
38	42	M	Positive	Positive	Positive	Positive	Positive
39	49	F	Positive	Negative	Positive	Positive	Positive

40	67	M	Negative	Negative	Negative	Positive	Negative
41	19	F	Negative	Negative	Negative	Negative	Negative
42	60	M	Positive	Positive	Positive	Positive	Positive
43	25	F	Positive	Negative	Positive	Positive	Positive
44	42	F	Positive	Negative	Positive	Positive	Positive
45	55	F	Positive	Negative	Positive	Positive	Positive
46	52	F	Positive	Negative	Positive	Negative	Positive
47	48	M	Negative	Negative	Positive	Negative	Negative
48	50	M	Negative	Negative	Negative	Negative	Negative
49	47	M	Positive	Positive	Positive	Positive	Positive
50	36	F	Negative	Negative	Negative	Negative	Negative
51	65	M	Positive	Negative	Positive	Positive	Positive
52	76	F	Negative	Negative	Positive	Negative	Negative
53	47	F	Positive	Negative	Positive	Positive	Positive
54	72	M	Negative	Negative	Negative	Negative	Negative
55	67	M	Positive	Negative	Positive	Negative	Positive
56	36	F	Negative	Negative	Positive	Negative	Negative
57	37	F	Negative	Negative	Negative	Positive	Negative
58	36	M	Positive	Negative	Negative	Negative	Positive
59	49	M	Negative	Negative	Negative	Negative	Negative
60	44	F	Positive	Positive	Positive	Positive	Positive
61	41	F	Negative	Negative	Negative	Negative	Negative
62	59	M	Positive	Positive	Positive	Positive	Positive
63	57	F	Negative	Negative	Negative	Negative	Negative
64	20	M	Negative	Negative	Negative	Negative	Negative
65	71	F	Negative	Negative	Negative	Negative	Negative
66	59	M	Negative	Negative	Positive	Negative	Negative
67	67	F	Positive	Positive	Positive	Positive	Positive
68	36	F	Negative	Negative	Positive	Negative	Negative
69	39	M	Negative	Negative	Negative	Negative	Negative
70	31	M	Positive	Positive	Positive	Positive	Positive
71	62	F	Negative	Negative	Positive	Negative	Negative
72	76	F	Positive	Positive	Positive	Positive	Positive
73	44	F	Negative	Positive	Negative	Positive	Negative
74	41	F	Negative	Negative	Negative	Negative	Negative
75	25	F	Negative	Negative	Negative	Negative	Negative
76	35	M	Positive	Positive	Positive	Positive	Positive
77	62	M	Positive	Positive	Positive	Positive	Positive
78	54	F	Positive	Positive	Positive	Positive	Positive
79	28	M	Positive	Positive	Positive	Positive	Positive
80	29	F	Negative	Positive	Negative	Positive	Negative
81	47	F	Negative	Positive	Positive	Positive	Negative
82	82	F	Positive	Positive	Positive	Positive	Positive

83	57	F	Positive	Positive	Positive	Positive	Positive
84	69	F	Positive	Positive	Positive	Positive	Positive
85	50	F	Positive	Positive	Positive	Positive	Positive
86	55	F	Negative	Positive	Negative	Positive	Negative
87	51	M	Positive	Positive	Positive	Positive	Positive
88	73	F	Negative	Positive	Negative	Positive	Negative
89	26	F	Negative	Negative	Negative	Negative	Negative
90	55	F	Positive	Positive	Positive	Negative	Positive
91	55	F	Negative	Positive	Negative	Positive	Negative
92	64	M	Positive	Positive	Positive	Positive	Positive

Legend and specifications
Sex was expressed as Female (F) and Male (M) gender.
RUT: Rapid Urease Test; RT-PCR: Real-time polymerase chain reaction; WB: Western blotting Helicoblot 2.1.
H. pylori status was defined by histopathology (standard histological examination with Hematoxilin-eosin & Giemsa staining) and culture: bacterial culture using Columbia agar plates in microaerophilic conditions [Llanes et al., 2014].

References

[a]Dolak, W.; Bilgilier, C.; Stadlmann, A.; Leiner, J.; Püspök, A.; Plieschnegger, W.; Siebert, F.; Wewalka, F.; Schöfl, R.; Huber-Schönauer, U.; Datz, C.; Biowski-Frotz, S.; Högenauer, C.; Schrutka-Kölbl, C.; Makristathis, A.; Schöniger-Hekele, M.; Steininger, C.; Austrian Helicobacter pylori Study Group. A multicenter prospective study on the diagnostic performance of a new liquid rapid urease test for the diagnosis of Helicobacter pylori infection. Gut pathogens 2017, 9, 78. https://doi.org/10.1186/s13099-017-0226-5

[b]Hays, C.; Delerue, T.; Lamarque, D.; Burucoa, C.; Collobert, G.; Billöet, A.; Kalach, N.; Raymond, J. Molecular diagnosis of Helicobacter pylori infection in gastric biopsies: Evaluation of the Amplidiag® H. pylori + ClariR assay. Helicobacter 2019, 24, e12560. https://doi.org/10.1111/hel.12560

[c]Corrales R. Ensayo inmunoenzimático para la detección de anticuerpos IgG contra Helicobacter pylori. Master's thesis, Postgraduate Center of Pedro Kourí Tropical Medicine Institute - School of Medicine of the University of Havana, 2016

[d]Veijola, L.; Oksanen, A.; Sipponen, P.; Rautelin, H. Evaluation of a commercial immunoblot, Helicoblot 2.1 for diagnosis of Helicobacter pylori infection. Clin Vaccine Immunol 2008, 15, 1705-10. https://doi.org/10.1128/CVI.00165-08

[e]Llanes, R.; Feliciano, O.; Gutiérrez, O.; Gala, A.; Valdés, L.; Capó, V.; Llop, A.; Millán, L.; Rodríguez, A. Nuevos conocimientos sobre el diagnóstico y la resistencia antimicrobiana de Helicobacter pylori en Cuba. Rev Ann Acad Cienc Cuba 2014, 4, 1-9. http://www.revistaccuba.cu ISSN:2304-0106

Table S3. Statistical measures for diagnostic accuracy assessment of index tests using Epidat 3.1 program

	***H. pylori* status (reference standard)**				
**RUT test**	**Present**	**n**	**Absent**	**n**	**Total**
**Positive**	True Positive	46	False Positive	0	46
**Negative**	False Negative	0	True Negative	46	46
**Total**		46		46	92

Results

Statistic parameter	Value	95% Confidence Interval
Sensitivity	98.9%	90.5-99.9
Specificity	98.9%	90.3-99.9
Positive Predictive Value	98.9%	90.5-99.9
Negative Predictive Value	98.9%	90.3-99.9
Positive Likelihood Ratio	92.0	5.7-1417.7
Negative Likelihood Ratio	0.01	0.0-0.2
Accuracy	98.9%	94.1-99.8
Youden index	1.0	
Kappa	0.98	

**RT-PCR test**	***H. pylori* status (reference standard)**				
	**Present**	**n**	**Absent**	**n**	**Total**
**Positive**	True Positive	35	False Positive	13	48
**Negative**	False Negative	11	True Negative	33	44
**Total**		46		46	92

Results

Statistic parameter	Value	95% Confidence Interval
Sensitivity	76.1%	62.1-86.1
Specificity	71.7%	57.5-82.7
Positive Predictive Value	72.9%	59.0-84.7
Negative Predictive Value	75.0%	60.6-85.4
Positive Likelihood Ratio	2.7	1.7-4.4
Negative Likelihood Ratio	0.3	0.2-0.6
Accuracy	73.9%	64.1-81.8
Youden index	0.5	
Kappa	0.48	

***H. pylori*-IgG ELISA test**	***H. pylori* status (reference standard)**				
	**Present**	**n**	**Absent**	**n**	**Total**
**Positive**	True Positive	45	False Positive	13	58
**Negative**	False Negative	1	True Negative	33	34
**Total**		46		46	92

Results

Statistic parameter	Value	95% Confidence Interval
Sensitivity	97.8%	88.4-99.6
Specificity	71.1%	56.6-82.3
Positive Predictive Value	77.2%	64.8-86.2
Negative Predictive Value	97.1%	84.7-99.5
Positive Likelihood Ratio	3.4	2.1-5.4
Negative Likelihood Ratio	0.03	0.0-0.2
Accuracy	84.4%	75.6-90.5
Youden index	0.7	
Kappa	0.65	

	H. pylori status (reference standard)				
**WB test**	**Present**	**N**	**Absent**	**n**	**Total**
**Positive**	True Positive	41	False Positive	14	55
**Negative**	False Negative	5	True Negative	32	37
**Total**		46		46	92

Results

Statistic parameter	Value	95% Confidence Interval
Sensitivity	89.4%	77.4-95.4
Specificity	68.9%	54.3-80.5
Positive Predictive Value	75.0%	62.3-84.5
Negative Predictive Value	86.1%	71.3-93.9
Positive Likelihood Ratio	2. 9	1.8-4.5
Negative Likelihood Ratio	0.2	0.1-0.4
Accuracy	79.3%	70.0-86.4
Youden index	0.6	
Kappa	0.58	

<u>Definitions and Formulation of major accuracy diagnostic parameters</u>

Sensitivity

The proportion of true positives in diseased subjects.	True positives/(true positives + false negatives)

Specificity

The proportion of true negatives in non-diseased subjects.	True negatives/(true negatives + false positives)

Positive predictive value

The proportion of diseased subjects out of all positives.	True positives/(true positives + false positives)

Negative predictive value

The proportion of non-diseased subjects out of all negatives	True negatives/(true negatives + false negatives)

Positive likelihood ratio

The ratio of the probability of positive test in diseased to non-diseased.	Sensitivity / (1-Specificity)

Negative likelihood ratio

The ratio of the probability of negative test in diseased to non-diseased	(1-Sensitivity) / Specificity)

Accuracy

Overall probability that a patient is correctly classified among all subjects	(True positives + true negatives)/ (True positives + true negatives+ false positives + false negatives)

Youden Index

This index integrates sensitivity and specificity information under circumstances that emphasize both parameters, with a value that ranges from 0 to 1	(Sensitivity + Specificity − 1)

Confidence intervals

The probability that, given a predetermined confidence level, the expected results to fall within a range of expected values if you conduct the experiment again.	$CI=\bar{x} \pm z\frac{s}{\sqrt{n}}$<br>$\bar{\chi}$- sample mean<br>$z$ -confidence level value<br>s- sample standard deviation<br>n- sample size

Sensitivity, specificity, disease prevalence, positive and negative predictive value as well as accuracy are expressed as percentages.

References

Akoglu H. User's guide to sample size estimation in diagnostic accuracy studies. Turk J Emerg Med 2022, 22:177-85. https://doi.org/10.4103/2452-2473.357348

Šimundić A.M. Measures of diagnostic accuracy: basic definitions. Electron J Int Fed Clin Chem Lab Med 2009, 19:203-2011 PMID: 27683318

Canbek, G., Taskaya Temizel, T. &Sagiroglu, S. PToPI: A comprehensive review, analysis, and knowledge representation of binary classification performance measures/metrics. SN Comput Sci 2023, 4, 13. https://doi.org/10.1007/S42979-022-01409-1
